# Effect of pellicle modification with polyphenol-rich solutions on enamel erosion and abrasion

**DOI:** 10.1590/1807-3107bor-2025.vol39.024

**Published:** 2025-02-21

**Authors:** Mariane Cintra MAILART, Ilirida BERISHA, Ana Sofia Arana REINALES, Samira Helena NIEMEYER, Alessandra Bühler BORGES, Tommy BAUMANN, Thiago Saads CARVALHO

**Affiliations:** (a) Universidade Estadual Paulista – Unesp, Institute of Science and Technology, Department of Restorative Dentistry, São Paulo, SP, Brazil.; (b) University of Bern, School of Dental Medicine, Department of Restorative, Preventive and Pediatric Dentistry, Bern, Switzerland.

**Keywords:** Fluorides, Polyphenols, Toothbrushing, Tooth Wear

## Abstract

The aim of the study was to compare the effect of salivary pellicle modification with polyphenol-rich solutions containing fluoride on enamel erosion and abrasion. Human enamel specimens (n = 14/group) were assigned to five pellicle-modifying groups: GSE+F (grape seed extract +500 ppm F^-^); CRA+F (cranberry extract +500 ppm F^-^); NaF (sodium fluoride solution -5 00ppm F^-^); Sn+F (commercial solution, SnCl_2_/NaF/AmF); and DW (deionized water, negative control). The specimens were submitted to 5 cycles, each one consisting of pellicle formation (120μl, 30 min, 37°C, no agitation), followed by pellicle modification with the experimental solutions (5 ml, 2 min, 25ºC, 70 rpm), and subsequent salivary pellicle formation (120 μl, 60 min, 37°C, no agitation). The specimens were then submitted to erosion (1% citric acid, 10 ml, 1 min, pH 3.6, 70 rpm, 25ºC). Subsequently, they were submitted to abrasion with a fluoride-based toothpaste slurry in a toothbrushing machine (50 strokes, 200 g load, 2 min exposed to slurry). The enamel surface was evaluated with an optical profilometer at baseline and after the 5 cycles to assess the surface loss. Data were submitted to Kruskal-Wallis followed by a multiple comparisons test (α = 0.05). Significant differences were found between the tested solutions (p <0.001). The highest surface loss was verified in the DW group (p < 0.001). The other tested solutions (GSE+F, CRA+F, Sn+F, NaF) promoted significant enamel protection against the erosive-abrasive challenges with no differences between them. In conclusion, the modification of salivary pellicle with both polyphenol-rich, commercial solution and fluoride solutions were able to protect the enamel surface from erosion and abrasion.

## Introduction

During a lifetime, teeth are exposed to several physical and chemical impacts, which might lead to tooth structure loss.^1^ Erosion occurs by the action of non-bacteria acids, which demineralize the teeth, making them more susceptible to wear. Abrasion arises from the friction of exogenous materials, for example during mastication or toothbrushing, forced over tooth substrates.^2^ In some cases the combination of these processes can result in erosive tooth wear (ETW).^2^ Indeed, the prevalence of this clinical condition has increased over the years, also in children and young adults. The prevalence in deciduous teeth ranges between 30% and 50% and in permanent teeth is between 20% and 45%.^3^ Nutritional factors and patient-related factors are crucial aspects responsible for the development of ETW.^6^ For example, the overconsumption of soft drinks and/or reflux or eating disorders can lead to erosive demineralization.

To minimize or prevent the progression of ETW many approaches to potentialize the protective effect of salivary pellicle have been studied. The pellicle is a thin acellular layer free of bacteria. It covers both hard and soft oral tissues and has a protective effect but cannot provide complete protection when long-term erosive and abrasive challenges occur.^7^ Natural compounds, such as polyphenols, can interact with and rapidly bind to proteins of the salivary pellicle.^8^ This increases the thickness of the pellicle and, in turn, its resistance to acid challenges.^8,9^ Several studies have shown the erosion-inhibiting potential of natural extracts with high contents of polyphenols such as grape seed extract and cranberry extract.^9^

Both grape seed and cranberry extracts contain proanthocyanidins. Although these polyphenols have shown high affinity with the salivary pellicle, the referred extracts behave differently. While the grape seed extract exhibited greater protective potential against erosion, the cranberry extract caused more demineralization when it was tested in its native pH.^9^ However, a previous study demonstrated that when the cranberry extract had its pH adjusted (to that similar as the grape seed extract), similar protective results were found.^10^ Furthermore, the addition of fluoride to these extract solutions have further enhanced their protective effect, showing a synergistic effect.^12^ Thus, these promising results justified testing the effect of these pellicle-modifying extracts in association with fluoride in an erosive model including additional abrasion of enamel. Accordingly, the present study investigated the protective effect of the modification of the pellicle with these fluoride/extract solutions on enamel erosion and abrasion. The following null hypothesis was formulated: the protective effect of salivary pellicle modified with the solutions is not different from negative control.

## Methods

### Study design

The present in vitro study followed a prospective and parallel group design with experimental solutions (independent variable) in five levels: GSE+F (grape seed extract + 500 ppm F^-^); CRA+F (cranberry extract + 500 ppm F^-^); Sn+F (commercial solution - Elmex™ - SnCl_2_/NaF/AmF); NaF (sodium fluoride solution – 500 ppm F^-^); DW (deionized water - negative control). The experimental unit was human enamel specimens, and the dependent variable was the surface loss (in µm).

### Specimen preparation

Enamel specimens (n = 70) were obtained from human molars in good condition. The teeth were selected from a biobank and no ethical approval was required as the local ethics committee (Kantonale Ethikkommision: KEK) categorizes the pooled biobanks as “irreversibly anonymized” samples. The crowns were separated from the roots and then sliced into vestibular and oral halves using an Isomet, 11-1180 Low-Speed Saw (Buehler, Düsseldorf, Germany). These halves were embedded in acrylic resin (Paladur, Heraeus Kulzer GmbH, Hanau, Germany) and serially ground under constant water cooling (Exakt 400 CS, Exakt, Norderstedt, Germany) with silicon carbide polyester film discs with decreasing grain size (15.3, 8.4, and 3 μm) leaving a smooth flat enamel surface. Between every grinding/polishing step, the specimens were sonicated for 5 min in deionized water. Thus, all prepared specimens had a flat ground enamel area with a 200-μm cut-off layer. The specimens were then stored in a saturated mineral solution (1.5 mM CaCl_2_, 1.0 mM KH_2_PO_4_, 50 mM NaCl, pH 7.0)^13^ until the start of the experimental procedures. Before the erosive-abrasive cycles, two thirds of the enamel surface were covered by adhesive tapes to be the reference area and the middle third was left exposed as it was the treatment area.

### Collection of pooled stimulated saliva

Volunteers (n = 8) in good general health, aged 23–62 years (mean age 36.5 years), and including both genders, donated stimulated saliva. Before the collection, the volunteers were instructed not to consume any food/beverage, except water, for at least 1 h. The saliva collection was carried out in the morning, between 9 and 10 am. The saliva production was stimulated by chewing a piece of paraffin for 10 min, and the whole saliva was collected in cooled flasks. Afterwards, the saliva was pooled and centrifuged for 15 min at 4 °C at 4,400 rpm. To avoid the degradation of salivary proteins, a protease inhibitor solution was added to the pooled saliva.^14,15^ Then, pooled saliva aliquots were stored at −80 °C until the start of the experiment. Because the saliva was pooled, the local ethical committee (Kantonale Ethikkommision: KEK) considers it as “irreversibly anonymized,” and no ethical approval is necessary.

### Preparation of the experimental solutions and their composition

Deionized water was used as negative control. The plant extract solutions (grape seed and cranberry) were formulated by individually dissolving the powder of the commercially available extracts in deionized water, then stirring for 30 min and filtering at room temperature. The extract solution was then mixed with NaF solution for a final fluoride concentration of 500 ppm and a final polyphenol concentration of 2 g/L, and pH was adjusted to 5.8.

A NaF solution was also prepared at 500 ppm F^-^ (pH adjusted to 4.5), and a commercial solution containing 800 ppm Sn^2^ and 500 ppm F^-^ (as AmF and NaF, pH 4.5) was used (Colgate-Palmolive, Świdnica).

### Erosion/abrasion cycle

The experiment comprised 5 cycles of erosion-abrasion, each consisting of pellicle formation with human saliva (120 μl, 30 min, 37°C, no agitation), followed by pellicle modification with experimental solutions (5 ml, 2 min, 25°C, 70 rpm), subsequent salivary pellicle formation (120 μl, 60 min, 37°C, no agitation) ^10^, and subsequently, the erosive and abrasive challenges were performed.

For erosion, the specimens were immersed in citric acid (1%, 10 ml, pH 3.6) at 25°C for 1 min, at 70 rpm. After rinsing with deionized water (20 s) and drying with air (5 s), the specimens were submitted to abrasion ^10^. The specimens were taken to an automatic brushing machine (RWTH Aachen University, Aachen, Germany), and freshly prepared toothpaste slurry was used for brushing. The slurry consisted of 1:1 (w/w) mixture of deionized water and fluoridated dentifrice (1450 ppm F^-^, Colgate Fresh Gel, Colgate-Palmolive, Grabetsmattweg, Therwil, Switzerland). The specimens were left in contact with the slurry for a total of 2 min, during which the brushing procedure was performed with 50 strokes (25 s) with a 200-g load using an American Dental Association (ADA) - approved toothbrush. After brushing, the specimens were again rinsed with deionized water and carefully dried for 5 s. All specimens were stored in a humid chamber between the cycles ^14^.

### Surface profilometry

To evaluate the surface loss (tooth wear), optical profilometry was performed considering an untreated reference area. Initially, a central enamel area of 3 x 1 mm was scanned, at a resolution of 10 x 20 µm with an optical profilometer (MicroProf 100 FRT GmbH, Bergisch-Gladbach, Germany). The initial height difference between reference and exposed areas was determined by subtracting the average height of the two outermost 0.5 x 1 mm areas (reference areas) from the average height of the central 0.5 x 1 mm area (exposed area), as determined using the analysis software of the profilometer, MARK III. Only specimens with height differences below 350 µm were selected for the study ^14^. After the final cycle, the tape-cover of the reference areas was removed, and the identical 3 x 1 mm area scanned again at the same resolution, and the height difference between reference and exposed areas determined similarly as explained above. Surface loss was then calculated as the change in height difference between reference areas and exposed areas from initial to final measurement.

### Statistical analysis

Data were submitted to the Shapiro-Wilk test, and they did not meet normal distribution (p < 0.001). Kruskal-Wallis followed by post-hoc multiple comparisons Dwass-Steel-Critchlow-Fligner tests were applied at a significance level of 5%. The statistical analyses were performed with Jamovi software version 2.3.19.

## Results

The median and interquartile range of enamel surface loss for each solution tested are depicted in Figure.

**Figure:**
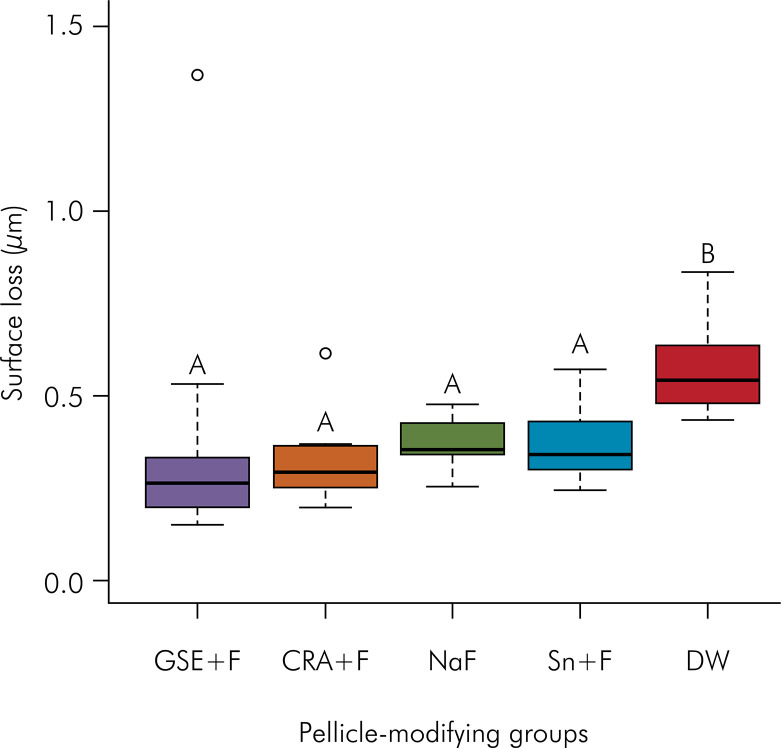
Enamel surface loss (µm) median values and interquartile range after erosive-abrasive challenges. Different capital letters mean significant differences between the groups.

Significant differences were found between the tested solutions (p < 0.001). Greatest erosive tooth wear (with highest surface loss) was verified for the DW group (negative control, p < 0.001). The other tested solutions used to modify the pellicle (GSE+F, CRA+F, Sn+F, NaF) were able to protect the enamel against the erosive-abrasive challenges, with significantly less surface loss than the negative control. There were no differences between the solutions.

## Discussion

Modifying the salivary pellicle with plant extracts has shown promising results in protecting enamel against tooth erosion.^8,9,11^ In the present study, grape seed and cranberry extracts were used in conjunction to fluoride to investigate the effect of salivary pellicle modification on erosion-abrasion challenge. These extracts are rich in polyphenols, which exhibit a high affinity for the proteins found in the enamel pellicle and significantly protect the enamel against erosive challenge.^8^ However, their protection potential has not been investigated yet when brushing is associated with the erosive challenge. The present study showed a significant protection of all the solutions tested, with significantly less enamel surface loss than the negative control, and thus, the null hypothesis was rejected.

The grape seed extract primarily consists of purified oligomeric proanthocyanidins (OPCs), which are generally procyanidin-type proanthocyanidins, and OPC more precisely strictly refers to dimers and trimers of catechins and epicatechins.^16^ In previous studies, these polyphenols showed potential for protecting enamel against erosion.^9^ Hen the grape seed extract solution was prepared at its natural pH of 5.8 without fluoride, it had already demonstrated remarkable results, according to previous studies. These included higher surface microhardness and surface reflection intensity compared to the other groups tested.^9^ The observed effects are possibly related to the interaction between the OPC and the basal layer of the salivary pellicle, leading to alterations in the pellicle morphology, such as an increase in its electron density and thickness.^9,17^ As previously investigated, this can be explained by hydrophobic interactions and hydrogen bonds resulting from the high affinity between OPC and salivary pellicle proteins, such as proline-rich proteins and statherin present in the basal layer of the pellicle.^12,18^

On the other hand, the cranberry extract solution prepared at its natural pH (3.2) caused more demineralization of the enamel, so, this solution was not able to protect the teeth against erosion.^9^ Cranberry extract also contains polyphenols similar to those found in grape seed extract such as anthocyanins, flavonols, proanthocyanidins, and tannis.^19^ A recent study using cranberry extract with the pH adjusted to 5.8 verified that the extract could protect enamel to a degree similar to grape seed extract.^10^ The regulation of pH mitigates the erosion caused by low pH levels, and the effect of these polyphenols on the pellicle was confirmed to be comparable to that observed with grape seed extract.^10^ Furthermore, it is important to highlight that only when fluoride (500 ppm) was added to the solutions, there was an effective protection against enamel erosion, and this protection was larger than the protection from fluoride alone. This finding was observed in our previous study, supporting the hypothesis that a synergistic effect between polyphenols and fluoride may contribute to erosion control.^10^

Other in vitro studies have also investigated these effects, also using different erosion protocols. However, when the erosion in combination with abrasion was tested, the plant extracts (GSE+F and CRA+F) and the solutions containing fluoride (Sn+F and NaF) similarly protected the enamel against erosive-abrasive processes. Fluoride is known to have a preventive role in dental erosion when higher-concentrated products are periodically used.^20,21^ When in contact with the enamel surface, fluoride interacts with hydroxyapatite, resulting in the formation of CaF_2 ._^21^ The CaF_2_ layer is assumed to function as a physical barrier with two modes of action, such as preventing acid contact with the underlying enamel and as a mineral reservoir that is targeted by erosive challenges. Consequently, after the acid exposition, the release of calcium and fluoride increases the saturation level of minerals in saliva, thereby promoting remineralization or preventing demineralization of the dental hard tissue. However, to be stable during frequent erosive challenges, the CaF_2_ layer may have to be dense enough, making this characteristic a limitation.^20,22^ Therefore, the ability of fluoride to protect enamel from this condition is somewhat restricted.

Due to its limited effect against erosion, promising alternatives to monovalent fluoride are available. Polyvalent metal ions have been investigated in several studies regarding their preventive effect on erosive demineralization. Tin (Sn, in the form of Sn^2^) or titanium (Ti, in the form of Ti^4^) in association with fluoride are the most common ions present in commercial mouthrinses.^21^ The Sn+F group was a commercial gold-standard solution to prevent the progression of erosive tooth wear. The tin in association with fluoride leads to the formation of a metal-rich surface with increased fluoride uptake and greater resistance to acids since the metal ion is incorporated into the enamel.^20,23^ The tin ions form different salts such as Sn_2_OHPO_4_, Sn_3_F_3_PO_4_, and Ca(SnF^3^)_2_ with the dental substrate, creating a stable layer. Furthermore, in addition to the formation of CaF^2^, stannous ions (Sn) can interact with tooth surfaces, leading to the formation of a so-called Sn-rich coating,^24^ characterized by deposits with an amorphous appearance distinct from that of CaF^2^.^25^ In eroded enamel, it was verified that tin deposition occurs on the tooth surface, with such ions being detected up to a depth of 20 μm in the enamel mineral.^25^ It has been hypothesized that fluoride and stannous ions in mouthrinses form deposits on the enamel surface, particularly with frequent exposure to acids and treatments, leading to a cumulative effect.^26^

Besides the interaction between the different compounds with hydroxyapatite, it is crucial to consider the presence of acquired enamel pellicle since the salivary proteins are fundamental in the development of erosion.^27^ In a previous study, a higher content of mucin, albumin, and carbonic anhydrase 6 was expressed in acquired enamel pellicle when it was treated with a rinse that combined Sn (800ppm/6.7mM, SnCl_2_) and F (225ppm/13mM, NaF).^28^ Mucin contributes to the maintenance of the acquired enamel pellicle structure, while albumin is responsible for adsorbing onto hydroxyapatite and penetrating enamel porosities.^28^ The carbonic anhydrase 6 is capable of neutralizing acids. On the other hand, the effect of sodium fluoride on acquired enamel pellicle leads to the expression of important salivary proteins. However, as the concentration of fluoride increases, the concentration of statherin and histatin 1 decreases.^29^ In contrast, the levels of basic proline-rich protein 2 increase with higher fluoride concentrations. This protein exhibited a high affinity to polyphenolic compounds, which can also explain the results of polyphenols-based solutions.

However, the Sn+F group presented no superior protection in comparison to the extract groups nor to the sodium fluoride solution. This might be associated with the mild erosive-abrasive model used in the present study. It has been suggested that severe challenges lead to a more pronounced protective effect of stannous ions, i.e., by a cumulative effect.^24^ Thus, considering the cumulative effect that is achieved with a high frequency of exposure to acid and treatments per day, it is suggested that severe models can better discern the treatment effects.^24^ Besides the erosion process, the abrasion also contributed to the enamel surface loss. The abrasion of dental hard tissues altered by erosion is considered the most significant interaction since it represents the clinical condition of ETW. In the present study, the abrasion model used a fluoride-based toothpaste, which simulates the intra-oral everyday clinical condition.^30^ It has been demonstrated that fluoride-based toothpastes induce less wear on eroded enamel both in vitro and in situ conditions ^31^. In this context, it is crucial to consider the mode of action of toothpaste slurries on eroded enamel. It has been suggested that short-term immersion of specimens in the slurry results in surface precipitation. Therefore, precipitation of CaF_2_-like material could be expected in in vitro studies, mainly because the slurry was prepared with a high content of fluoride toothpaste.^32^ Moreover, it has been hypothesized that softened enamel can be removed even by weak forces, such as friction from oral soft tissues, and even more so after stronger impacts.^33,34^ It has already been demonstrated that abrasive challenges partially remove the altered superficial prism layer, exposing a slightly harder underlying enamel layer.^34,35^ This could explain why there was no difference between the tested experimental solutions submitted to the erosive-abrasive challenges.

Although no differences were observed, we can predict from the pattern on the graphs that the extract solutions had slightly less surface loss than the fluoride groups. We used in the present study a very initial model, with only 5 erosion cycles different from other more severe models tested.^26,36,37^ In addition, this initial model may explain the low surface loss values observed, which were close to the detection limit of the optical profilometer and represent a limitation of our study. We speculate that with more severe protocols to be carried on, there possibly might be differences between our experimental extract solutions. It is essential to consider clinical conditions of salivary pellicle formation to ascertain the efficacy of the polyphenol-based solution in protecting against enamel erosive wear. Modifying salivary pellicle with grape seed and cranberry extracts containing fluoride is a promising option for the prevention of erosive tooth wear. However, further studies are required not only to evaluate long-term effects but also severe erosive conditions should be tested.

## Conclusion

The modification of salivary pellicle with polyphenol-rich solution, commercial and fluoride solutions were able to protect the enamel from erosion and abrasion.
