# Erratum

**DOI:** 10.1590/1807-3107bor-2025.vol39.024rr

**Published:** 2025-03-14

In article Effect of pellicle modification with polyphenol-rich solutions on enamel erosion and abrasion, with DOI number https://doi.org/10.1590/1807-3107bor-2025.vol39.024, published in the journal Brazilian Oral Research, v. 39, e024, 2025, on p. 6, Acknowledgment

**Where it read:**

Ilirida Nikolla

**Read:**

Ilirida Berisha

